# Contemporary Approaches to Postpartum Hemorrhage with Focus on Uterine Compression Sutures—An Updated Comprehensive Systematic Review

**DOI:** 10.3390/medsci14060619

**Published:** 2026-09-30

**Authors:** Alexandra Ursache, Demetra-Gabriela Socolov, Iuliana-Elena Bujor, Cristina-Elena Mandici, Maria-Elena Nita, Monica Titianu, Carmen Pintilescu, Iustina Petra Solomon-Condriuc, Ecaterina Tomaziu-Todosia Anton, Alexandru Carauleanu, Daniela Roxana Matasariu

**Affiliations:** 1 Grigore T. Popa University of Medicine and Pharmacy Iasi, 700115 Iasi, Romania; alexandra.ursache@umfiasi.ro (A.U.); demetra.socolov@umfiasi.ro (D.-G.S.); iuliana_elena.bujor@umfiasi.ro (I.-E.B.); cristina-elena.mandici@umfiasi.ro (C.-E.M.); nita.maria-elena@d.umfiasi.ro (M.-E.N.); iustina-petra.solomon@umfiasi.ro (I.P.S.-C.); tomaziu-todosia.ecaterina@d.umfiasi.ro (E.T.-T.A.); daniela.matasariu@umfiasi.ro (D.R.M.); 2 Department of Obstetrics and Gynecology, Cuza Voda Hospital, 700038 Iasi, Romania; moni_titianu@yahoo.com; 3 Faculty of Economics and Business Administration, Alexandru Ioan Cuza University of Iasi, 700505 Iasi, Romania; carmen.pintilescu@uaic.ro

**Keywords:** postpartum hemorrhage, obstetric hemorrhage, B Lynch, compression suture, Hayman suture, Cho suture, arterial ligation, PPH surgical management

## Abstract

**Background/Objectives**: Surgical management of postpartum hemorrhage (PPH) is essential when conservative measures fail, yet no clear hierarchy of effectiveness among interventions exists. Given the scarcity of the direct comparative studies and the substantial clinical and methodological heterogenicity of the available evidence, this systematic review aims to provide an updated and structured synthesis of the UCS for the management of refractory PPH. **Methods**: We reviewed studies published from January 2014 to December 2025 using keywords related to PPH and UCS. After excluding duplicates and applying the inclusion and exclusion criteria, we selected 31 articles. **Results**: We grouped the selected articles into 4 sections: UCS alone; UCS versus uterine artery embolization (UAE) and versus Bakri balloon tamponade; newer UCS techniques; and articles on future fertility outcomes after UCS. Evidence on UCS alone was predominantly uncontrolled observational case series, with reported efficacy ranging from 73.9% to 100%. Newer UCS techniques use removable sutures to reduce side effects and complications. Although early results on subsequent fertility and menstrual function appear promising, the lack of large, well-designed randomized studies warrants caution in interpretation. **Conclusions**: Asian tertiary centers dominate the literature and report high uterine preservation rates, but retrospective design, small samples, lack of controls, inadequate adjustment for confounders, and heterogeneous techniques limit the strength of conclusions and generalizability. The predominance of cesarean section cohort further limits generalizability to vaginal births. UCS efficacy appears strongly influenced by bleeding severity and case complexity.

## 1. Introduction

Postpartum hemorrhage (PPH) is diagnosed when blood loss exceeds 500 mL after vaginal delivery or 1000 mL after cesarean section. However, these volumetric thresholds are often unreliable because visual estimation tends to underestimate actual blood loss [1]. To address these limitations, several organizations have refined the diagnostic criteria [2,3]. The American College of Obstetricians and Gynecologists (ACOG) define PPH as blood loss exceeding 1000 mL accompanied by signs or symptoms of hypovolemia within 24 h of birth, irrespective of the mode of delivery [4]. The Royal College of Obstetricians and Gynecologists (RCOG) has categorized PPH by severity [5], and the World Health Organization (WHO) still uses the traditional thresholds of more than 500 mL (vaginal) and more than 1000 mL (cesarean), sometimes further categorizing severity as mild, moderate, and severe [6].

PPH prevalence ranges from 6% to 11% globally, with the highest burden in low-resource countries. PPH remains the leading cause of maternal morbidity and mortality, with a maternal mortality risk of 16.67% in developing countries [7]. Risk factors are often absent, though clinicians use the “four Ts” to identify the most common etiologies: uterine atony (70–80% of PPH cases); genital tract lacerations or rupture; retained products of conception; and coagulopathies. Other contributors include abnormal placentation, uterine inversion, prolonged labor, multiparity, polyhydramnios, macrosomia, chorioamnionitis, and previous instrumentation [5,8,9]. In the context of increasing demand for cord blood collection, a 2021 research by Mlodawski et al. underlines the risk of this maneuver in prolonging the third stage of delivery and increasing the risk of blood loss in women [10].

The adverse consequences of PPH extend beyond mortality and include shock, transfusion-related complications, multi-organ failure, thrombosis, sepsis, prolonged intensive care unit (ICU) stays, and infertility. Many of these outcomes are preventable with timely recognition and aggressive management [1].

Management typically progresses from conservative to invasive strategies. First-line interventions include uterotonics. Non-pharmacological options such as uterine massage, bimanual compression, aortic compression, and balloon tamponade are essential, with escalation to surgical techniques such as compression sutures (B Lynch, Hayman, Cho), vessel ligation, embolization, and hysterectomy when conservative measures fail [2,4,6]. Although the success rate (SR) of these conservative surgical techniques exceeds 75%, there are reports of related complications, including hematometra (intrauterine blood collection), pyometra (intrauterine purulent collection), and uterine ischemia [11].

On the preventive side, active management of the third stage of labor with prophylactic uterotonics (preferably oxytocin) is universally recommended by FIGO (2012), WHO (2018), ACOG (2017), and others [2,4,5,6]. Despite abundant guidelines for PPH management, disparities persist because of differences in resources, training, and implementation, underscoring the need for strategies tailored to institutional and regional capacity to bridge the gap between guidelines and practice.

Surgical management of PPH is a critical intervention when conservative measures fail; however, there is no clear hierarchy of effectiveness among available surgical interventions, including compression sutures, vessel ligation, embolization, or hysterectomy, and it remains unclear which yields the best outcomes in different clinical contexts. The underling evidence remains dominated by small, retrospective, uncontrolled case series, with substantial heterogenicity in patient selection, PPH etiology and severity, UCS techniques, the definition of hemostatic success, concomitant interventions, and follow-up duration. Most recommendations are therefore based on case series, observational reports, or expert opinion rather than high-quality evidence. Given the scarcity of the direct comparative studies and the substantial clinical and methodological heterogenicity of the available evidence, this systematic review aims to provide an updated and structured synthesis of the UCS for the management of refractory PPH, as other conservative methods have already been extensively studied and are well established in guidelines and systematic reviews.

## 2. Materials and Methods

### 2.1. Research Strategy

ECM and IEB, two evaluators, independently reviewed the literature for significant studies published between January 2014 and December 2025. To reach consensus, discrepancies were discussed with a third reviewer (DRM). We used all possible combinations of keywords and phrases to search the Cochrane Central Register of Controlled Trials (CENTRAL), Web of Science, and PubMed/Internet portal of the National Library of Medicine. This review was performed in accordance to the PRISMA (Preferred Reporting Items for Systematic Reviews and Meta-Analyses) guidelines. This Systematic Review has not been registered on platforms such as PROSPERO.

**Figure 1:**
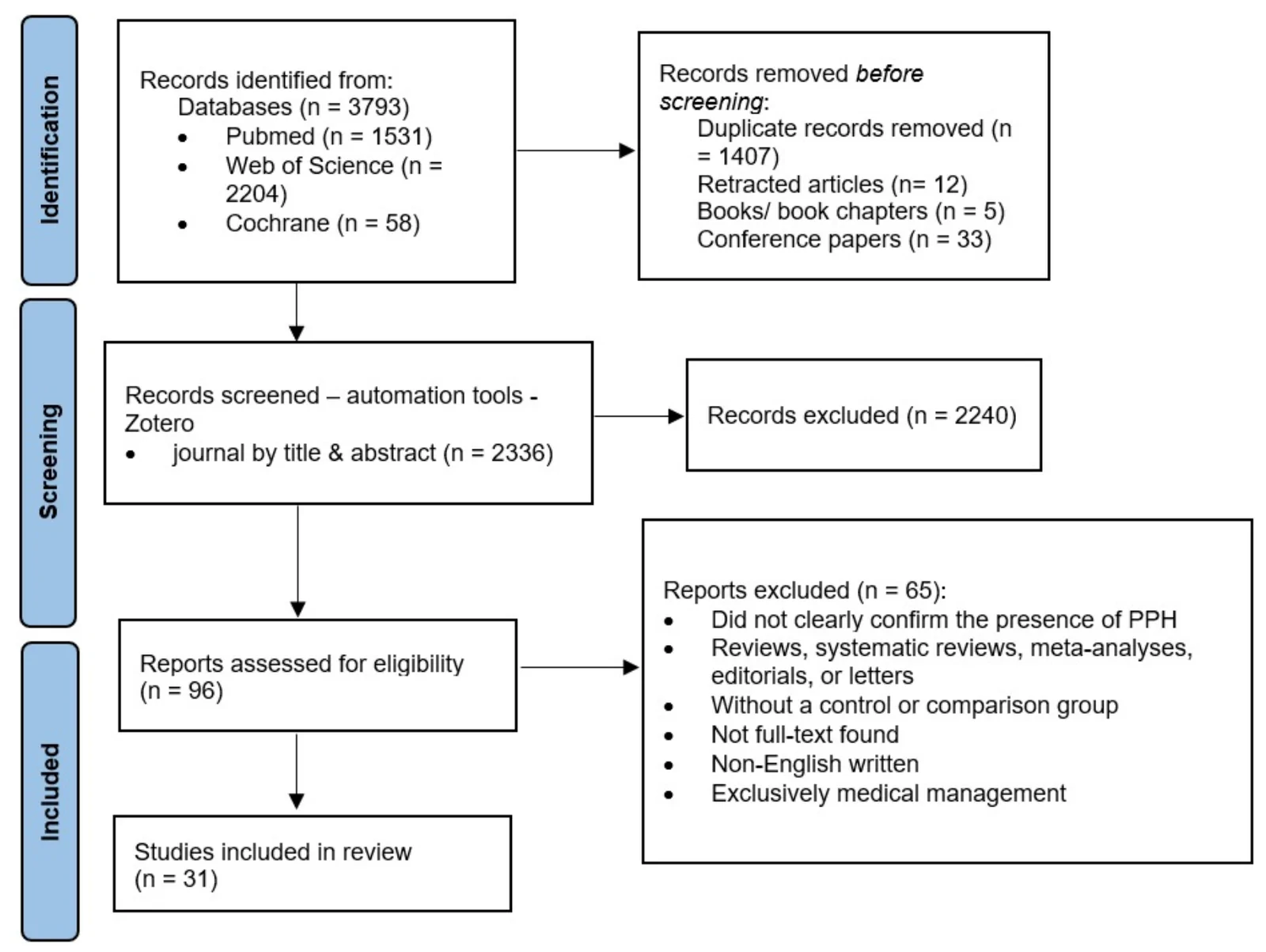
PRISMA 2020 flow diagram of study selection (Preferred Reporting Items for Systematic Reviews and Meta-Analyses).

The terms included “postpartum hemorrhage (PPH)”, “obstetric hemorrhage”, “postpartum bleeding”, “PPH surgical management”, “peripartum hysterectomy”, “artery/arterial ligation”, “uterine devascularization”, “B Lynch/B Lynch suture/B Lynch”, “compression suture/uterine compression suture”, “Hayman suture”, “Cho suture”, “PPH surgery”, and “laparotomy” (Figure 1, Table 1).

### 2.2. Dissemination and Ethics

For our review of surgical management of postpartum hemorrhage, ethical approval is not required.

### 2.3. Eligibility Criteria

Inclusion criteria. Studies were considered eligible for inclusion in this review if they met one or more of the following criteria: (1) included women diagnosed with PPH who required surgical or interventional management; (2) reported original data from case reports, observational studies (cohort or case-control); (3) were human studies; (4) included a comparison group (either women with PPH managed non-surgically or women without PPH) or did not include one; (5) provided comparative data on UCS vs. other surgical techniques or radiological intervention for PPH (such as artery embolization, arterial ligation, balloon tamponade); (6) were published as full-text articles in any language.

Exclusion criteria. Studies were excluded if they met any of the following criteria: (1) did not clearly confirm PPH or the need for surgical intervention; (2) lacked sufficient details on participants or interventions; (3) were animal, in vitro, or laboratory-based studies; (4) were reviews, systematic reviews, meta-analyses, editorials, or letters; (5) focused exclusively on medical management; and (6) were available only as abstracts, not in full text.

### 2.4. Quality Assessment

Two independent reviewers assessed the risk of bias in the included studies using the Cochrane Risk of Bias tool for randomized controlled trials or the Newcastle-Ottawa Scale for non-randomized studies. Any disagreements were resolved through discussion with a third reviewer.

## 3. Results

Research using the above-mentioned keywords identified 112 articles in PubMed, 225 in Web of Science, and 26 in the Cochrane Central Register. After excluding 53 duplicate articles and applying the inclusion and exclusion criteria, we selected 31 articles. The flow chart of our selection process is shown in Figure 1.

We then grouped the selected articles into 4 sections: those about UCS alone; UCS versus other conservative techniques (uterine artery embolization (UAE) and Bakri balloon tamponade); newer types of UCS; and articles on future fertility outcomes after UCS and on research presenting UCS complications.

A. Uterine compression sutures (UCS)

**Figure 2:**
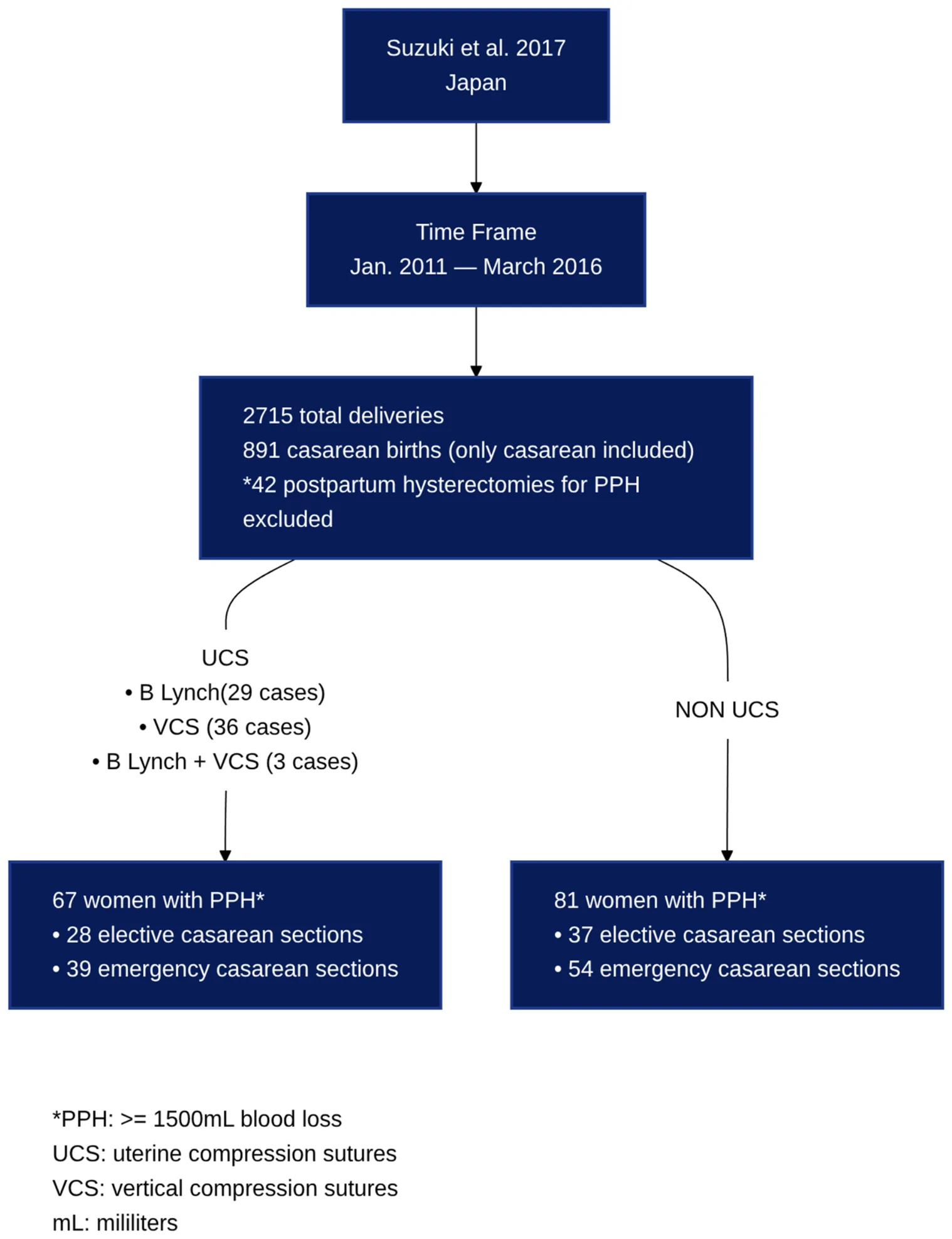
Suzuki et al. research—Investigation of perioperative complications associated with use of uterine compression sutures [12].

**Figure 3:**
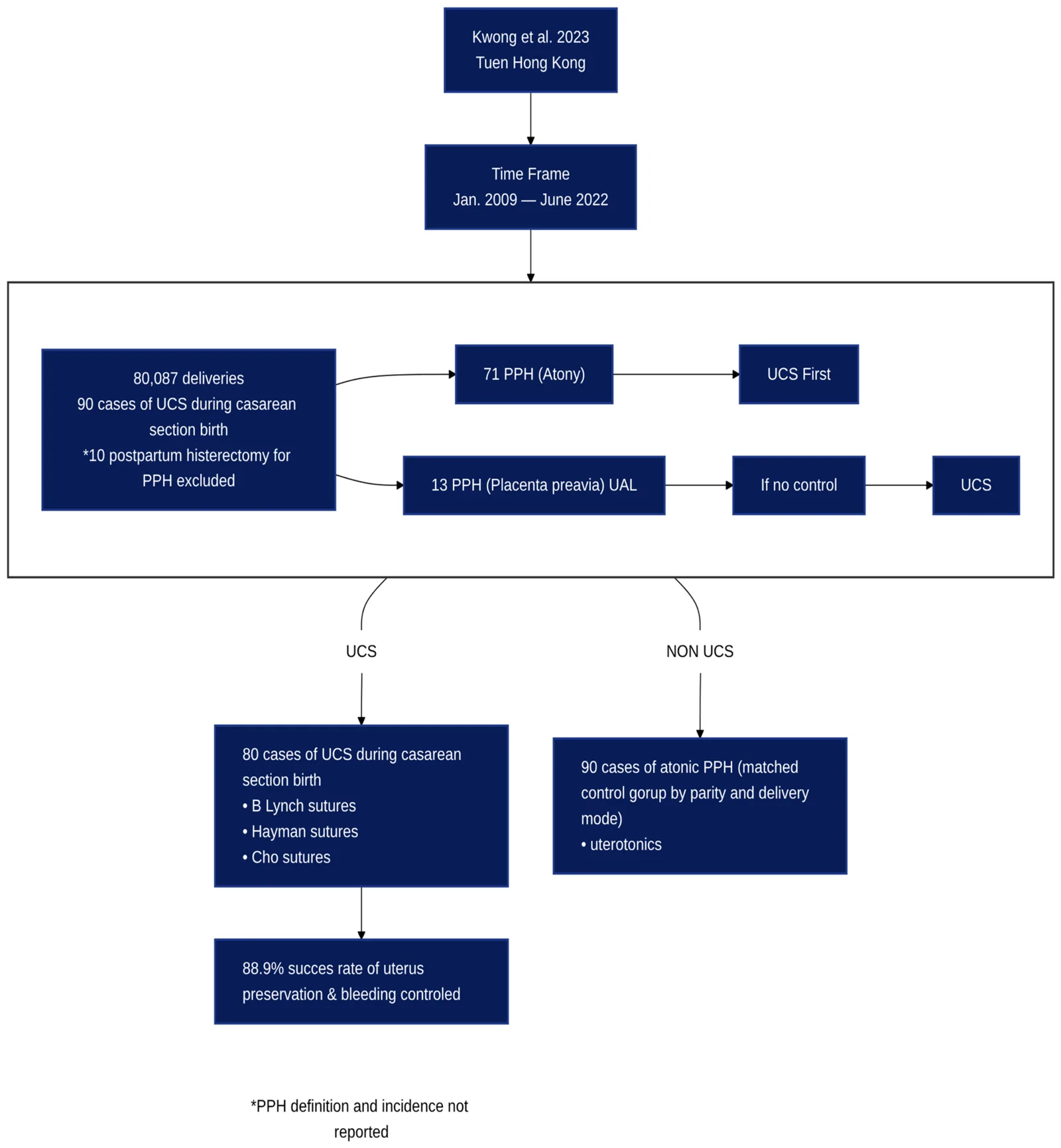
2023 Kwong et al. research—Menstrual, fertility and psychological impacts after uterine compression sutures for postpartum hemorrhage: a prospective cohort study [8].

Of the eight studies reviewed in this category [8,11,12,13,14,15,16,17], only Suzuki et al. (2017) [12] and Kwong et al. (2023) [8] provide moderate- to low-level evidence (Figure 2 and Figure 3) [8,12]. The remaining literature is dominated by level IV retrospective case series (Table 2), with Charoendumrongsup et al. (2015) having the weakest contribution to the PPH literature [14].

B. Uterine Compression Sutures vs. Bakri balloon tamponade, UAE, or other conservative surgical methods

**Figure 4:**
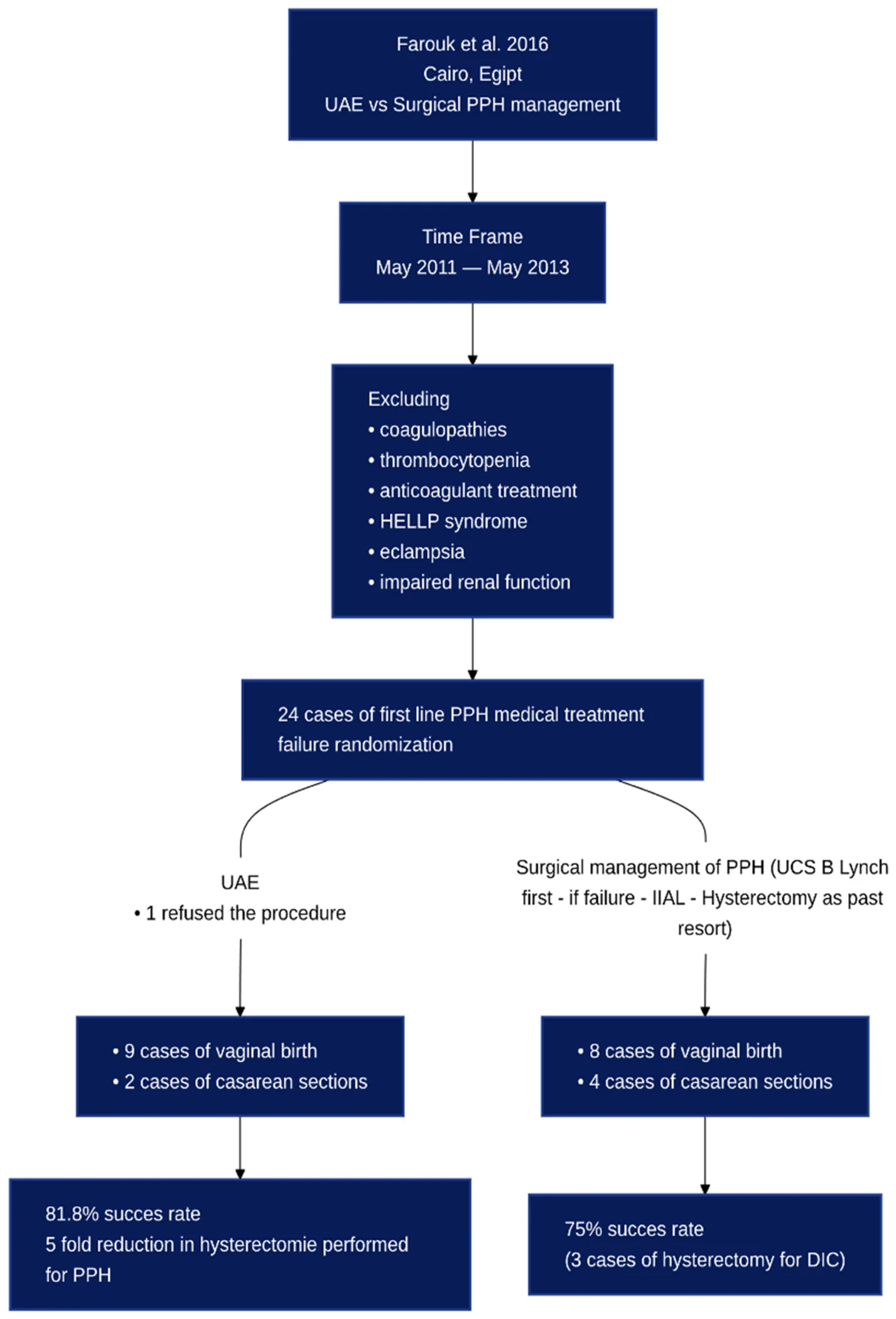
Farouk et al.—Uterine artery embolization versus surgical management in primary atonic postpartum hemorrhage: A randomized clinical trial [18].

Four studies compared uterine-sparing techniques for PPH refractory to uterotonics [18,19,20,21]. A small Egyptian randomized controlled trial by Farouk et al. in 2016 [18] found that stepwise devascularization plus B Lynch sutures with Vicryl No. 1 was successful in 75% of cases, compared with 81.8% for UAE, and that the hysterectomy rate after embolization was fivefold lower (Figure 4) [18].

**Figure 5:**
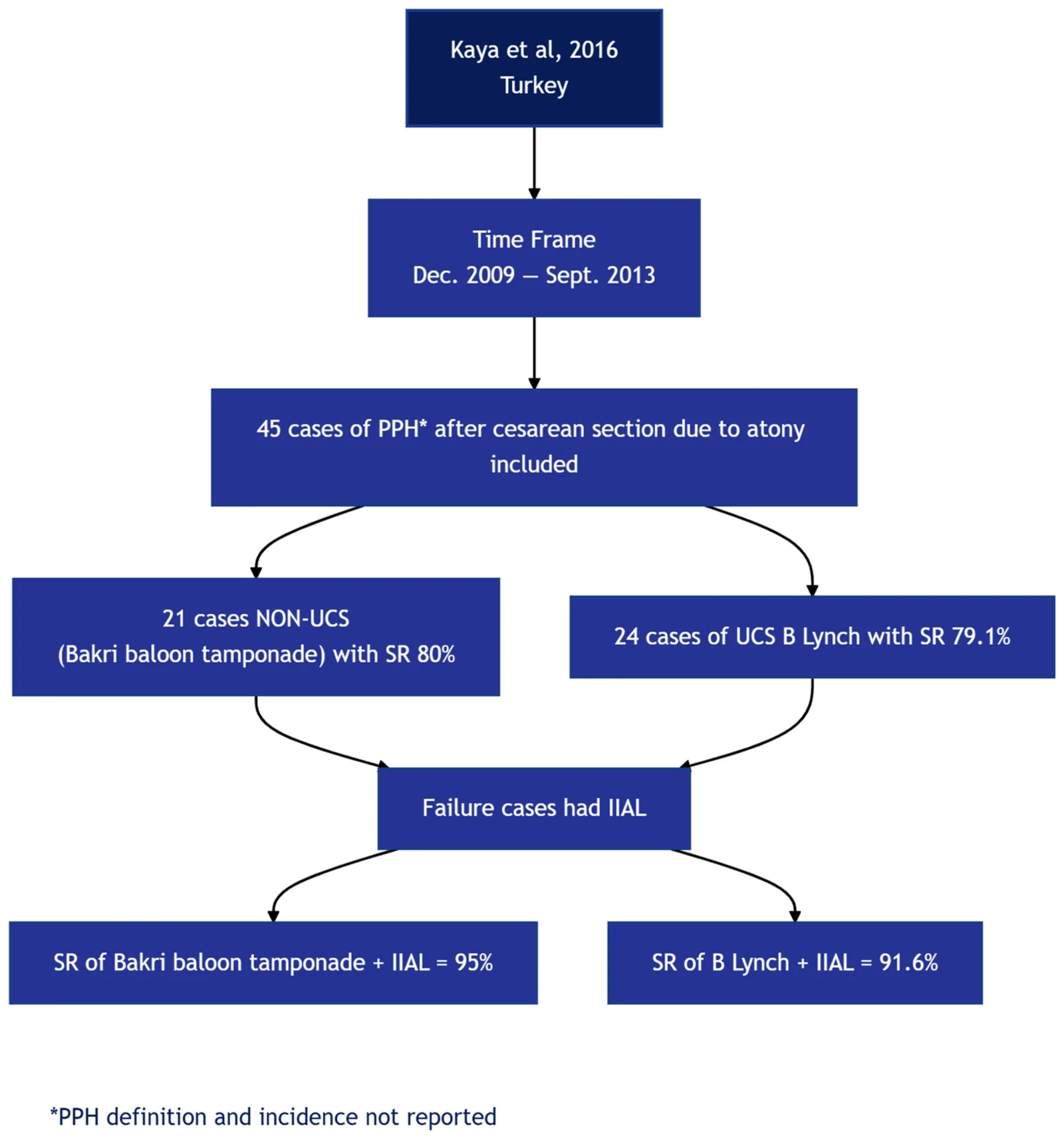
Kaya et al.—Which uterine sparing technique should be used for uterine atony during cesarean section? The Bakri balloon or the B Lynch suture? [19].

A Turkish retrospective comparison by Kaya et al. in 2016 reported similar success rates (SR) for classical B Lynch sutures (79.1%) and Bakri balloon tamponade (80%), with success rates exceeding 90% when IIAL was added (Figure 5) [19].

**Figure 6:**
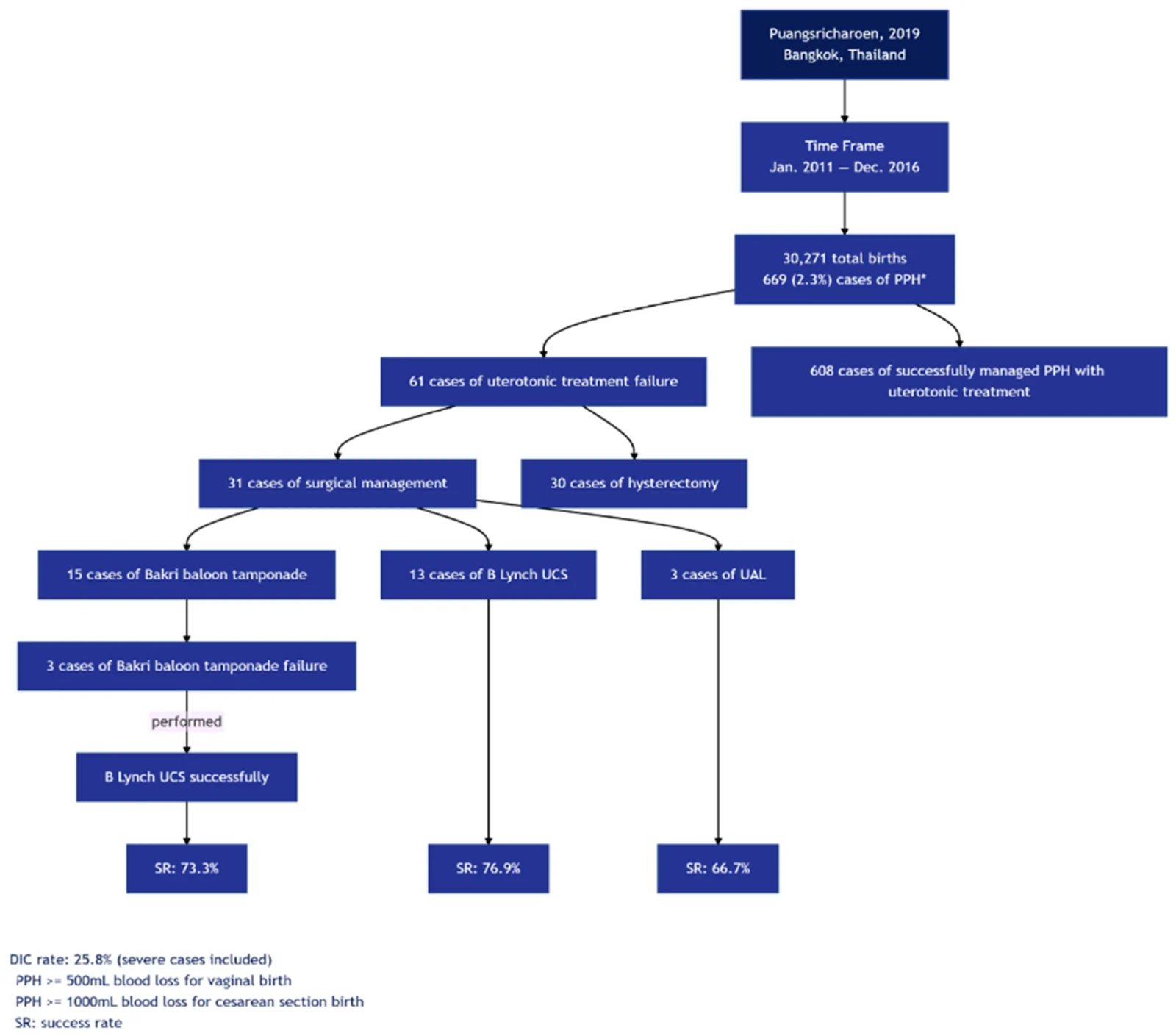
Puangsricharoen et al.—Conservative surgical management for immediate postpartum hemorrhage [19].

In a Bangkok series of 30,271 births analyzed by Puangsricharoen et al. in 2019 [20], with a 2.3% PPH incidence, conservative surgery after failed uterotonics succeeded in roughly two-thirds of cases, with success rates of 66.7% for Bakri balloon tamponade, 75% for B Lynch, and 66.7% for UAL (Figure 6) [20].

**Figure 7:**
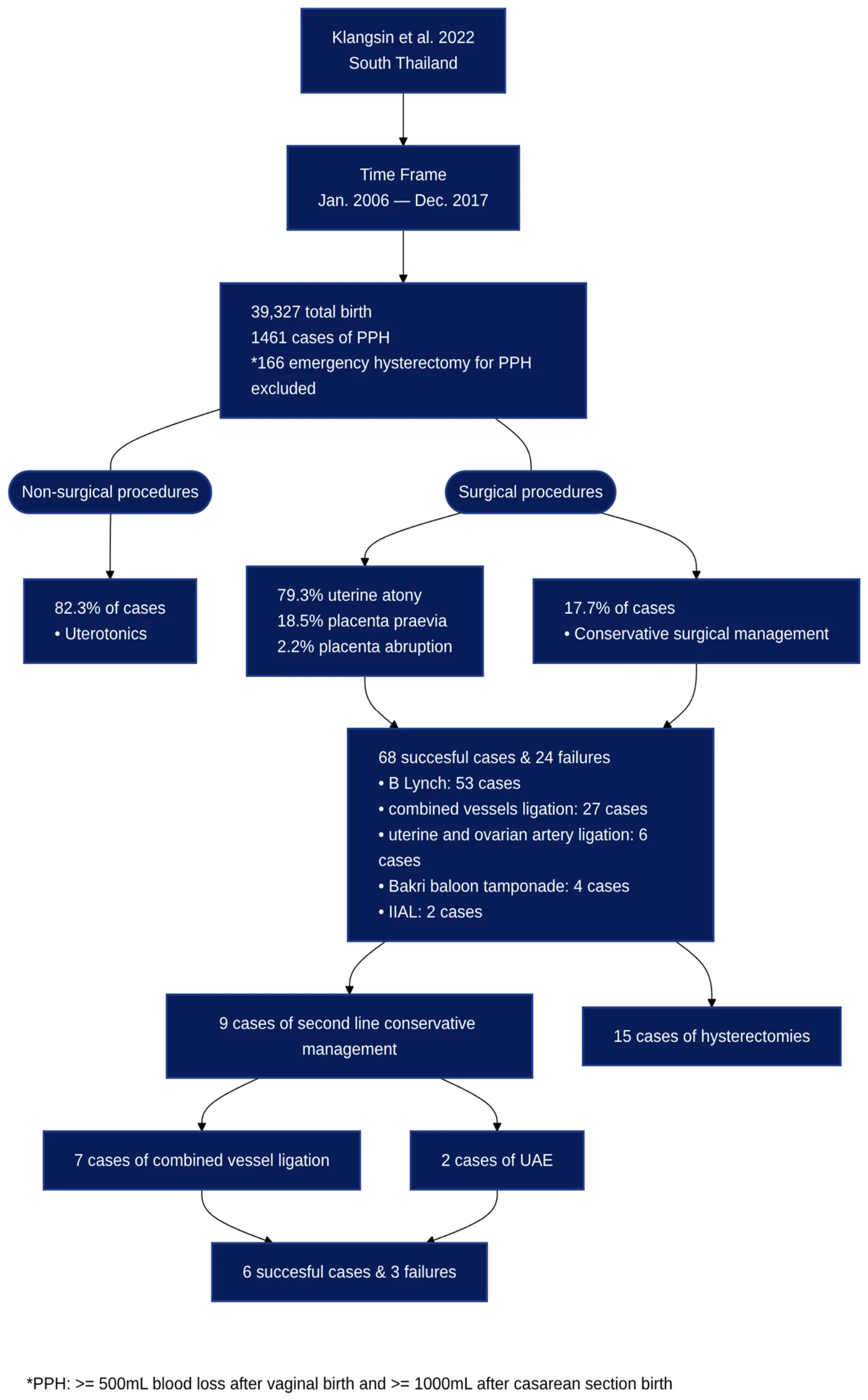
Klangsin et al.—Conservative Surgical Management of Early Postpartum Hemorrhage: A 12-Year Experience in a Tertiary Care Center in the South of Thailand [21].

A larger 12-year study published by Klangsin et al. in 2022 [21], comprising 39,327 births, reported a 3.7% early PPH rate, achieved 73.9–80.4% success with conservative surgery, mainly B Lynch, and reported a 19.6% hysterectomy rate and a 27.2% early-complication rate dominated by DIC and organ failure (Figure 7) [21].

C. Newer types of removable UCS

A retrospective case series from China, conducted from January 2013 to December 2016, included 12 women with PPH during cesarean section who failed conservative treatment. The authors devised a new removable utero-pubic ring compression suture using No. 1 Vicryl with a round needle, passed through the vagina and removed after 48 h. The uterus is brought out of the abdominal cavity. The first stitch starts on the posterior uterine wall approximately 0.5 cm below the attachment of the right uterosacral ligament, and the needle is inserted vertically from the posterior wall into the uterine cavity. Another similar suture is performed on the left side. The two upper ends are knotted together inside the uterine cavity, and the 30 cm free ends are pushed downward through the cervical canal into the vagina. After closure of the hysterotomy, the caudal lower end of the right suture is passed through the right lower peritoneum and abdominal wall, exiting externally 2 cm lateral to the midline and a cm above the symphysis pubis, with the left suture mirroring the same technique. The caudal ends from the abdominal wall and the cephalic ends pulled from the vagina are tied tightly together, creating a complete ring that binds the uterus beneath and behind the pubic bone—“ring compression suture”. The threads are passed through a latex F-28 pipe to prevent cutting into the skin or vaginal mucosa. The uterus is compressed against the pubic bone in an anteflexed position, combining intrauterine, vaginal, and abdominal wall components to form a ring. The suture is removed after 48 h. The women’s ages ranged from 22 to 36 years, with 4 multiparous and 8 nulliparous, and a mean fetal birth weight of 4005 g. Atonia reported causes were: 4 cases with macrosomia, 3 with polyhydramnios, 3 with prolonged labor, and 2 pregnancies with placenta previa. The mean blood loss in these cases was 1383 ± 295 mL and the mean blood transfusion 367 ± 239 mL. The authors report a 100% success rate, with no hysterectomy cases and a mean follow-up time frame of 31.1 ± 17.9 months. Normal postpartum menstrual flow, with menstruation returning in 2–5 months, and 2 successful subsequent pregnancies. Minor limitation—the distal vaginal side of the cervix cannot be compressed, but bleeding from there is rare. It is a single center retrospective study, with no control group, with a small sample size, having a medium follow-up for fertility outcomes, and presenting no data on the overall incidence of PPH, total number of births, or type of health care center [22].

A retrospective case series with no control group, conducted at a hospital affiliated with Capital Medical University in Beijing, China, from January 2010 to January 2019 (9-year time frame), included 39 women with atonic PPH during cesarean section who failed conservative management. Management involved placing a Vicryl No. 1 polyglactin absorbable braided compressive suture in an anteflexed position against the pubic bone, removed after 48 h. The suture began at the posterior uterine wall at the level of the right uterosacral ligament. The needle was inserted vertically from the posterior wall into the uterine cavity, then passed through the eye of a No. F-30 drainage tube, exiting the posterior wall on the left side at the same horizontal level. After closing the cesarean section incision, the needle was passed through the left lower peritoneum and exited the abdominal wall 1 cm lateral to the midline and 1 cm above the symphysis pubis, approximately 2–3 cm below the cesarean skin incision. Another suture was passed on the contralateral side symmetrically, exiting on the right lower abdomen. The uterus was placed in marked anteflexion and compressed against the lower abdominal wall and the pubis by an assistant, with both ends of the suture tied under moderate tension. The suture was removed after 48 h. The drainage tube, with any remnant threads, was easily removed through the vagina. The women’s ages ranged from 23–37 years with 16 of them being nulliparous and 23 multiparous. The mean gestational age was 39 weeks 1/7, and the mean fetal birth weight was 4132 ± 424 g. The underlined risk factors for atonia included 16 prolonged labors, 15 macrosomia cases, 7 polyhydramnios cases, and 1 twin pregnancy. The mean blood loss in these cases was 1379 ± 228, with blood transfusion ranging from 0–600 mL. The success rate was 92.31%. Authors report 3 cases with failure that were treated with additional uterine artery ligation. They report no cases of hysterectomy, and no postoperative complications such as ischemia, necrosis, intrauterine synechiae, infection, or anatomical/physiological abnormalities. Mean time to resumption of menstruation was 8.37 months postpartum. No lower abdominal pain was reported by the included women. The article did not report the total number of births or the incidence of PPH. It is level 4 evidence, a retrospective case series with a small sample and no control group. It took place in two health care facilities, with potential selection bias, a limited long-term fertility assessment, and no data on subsequent fertility rate [23].

The article represents a prospective cohort study with systematic data collection and statistical analysis, conducted at a provincial hospital affiliated with Shandong University in Jinan, China, without an explicitly defined time frame. It focuses on technique development and application rather than a consecutive series over specific years. Women with severe PPH were included if conservative measures failed during or after cesarean section. Two types of UCS were used, with 2 cases using modified B Lynch sutures and 3 using Hayman suture. UCS were made using a 2-polyglactin Vicryl-type braided absorbable suture, with no further specification. The sutures are designed to be removed through the vagina after a mean of 21.6 h. A number 2-Polyglactin Vicryl suture is used. The suture is passed through the uterine cavity in a classic B Lynch pattern, starting from the lower anterior right side and reaching the upper anterior left side, crossing over the uterine fundus to the posterior wall. A lose 7-silk thread is also placed in the cavity as a marker, and the free ends are brought out through the cervix into the vagina for later access. A 1 cm cannula is used to remove the sutures after the risk of hemorrhage passes. The knots are cut inside the cannula, with the posterior part cut first, followed by removal of the anterior part. The removable Hayman suture consists of a simple vertical brace suture, with no horizontal fundal crossing. The sutures pass from the anterior lower segment of the uterus, over the uterine fundus, to the posterior uterine wall. Their tails are brought out into the vagina. After the acute risk has passed, a suction cannula of 1 cm diameter is used to remove the sutures. The vaginal thread is pulled into the suction cannula until the knot is inside it. The knot is cut inside the cannula, and afterward the suture is gently pulled out. The article does not provide data on patient and delivery characteristics such as age, parity, or blood loss volume. The success rate was 100%, with no immediate or short-term complications reported. Data are also missing on the total number of deliveries per year or during the study period, the overall incidence of PPH in the institution, and details about the PPH definition, blood loss, or transfusion necessity. There is no comparison between the two types of compression sutures used, and follow-up data are limited (fertility, menstruation, subsequent pregnancies). It is a single-center study, subject to publication bias, still requires vaginal access for removal, and may not be suitable in all clinical scenarios [24].

It is a retrospective case series conducted in Marrakech, Morocco, from 2006 to 2011. 15 women with severe PPH were included, all of whom failed conservative management with uterotonics and vascular ligature. The authors present a novel non-absorbable, removable uterine brace, a No. 2 sliding round-needle compressive suture that compresses the uterus against the pubic bone. The suture is inserted from the outside through the anterior abdominal wall, 2 cm above the pubis and 2 cm lateral to the midline, then passed through the lower uterine segment from the anterior to the posterior wall as low as possible, below the hysterotomy incision and 2 cm medial to the uterine artery. The suture is then passed over the uterine fundus like a brace, compressing it approximately 3–4 cm inside the cornual border, and the needle exits through the anterior abdominal wall 2 cm above the first stitch but 4 cm lateral to the midline. The procedure is repeated symmetrically on the opposite side. The lower ends are tied anteriorly, and the upper ends are tied with additional curves. The suture is removed percutaneously within 24–48 h. The mean maternal age was 25 years, with 12 primiparous and 3 at their second births. There were eleven vaginal deliveries and 4 cesarean sections. The main causes of PPH were uterine atony in 80% of cases, placenta accreta in 13%, and uterine rupture in 7%, with a mean estimated blood loss of 2300 ± 500 mL. The initial reported efficacy rate of the method was 100%, with one secondary hysterectomy due to bleeding relapse after 3 h and one maternal death due to preeclampsia with a cerebrovascular accident not directly related to the suture technique. Long-term outcomes were reported, with normal menstrual cycles in all women and 3 of 5 women who attempted subsequent pregnancies achieving pregnancy. No data were provided on the total number of births per year, the overall incidence or percentage of PPH, or the proportion managed with this suture. The only definition of PPH provided in the article was uncontrolled bleeding persisting after initial uterotonic management, with hemodynamic instability despite resuscitation with crystalloids and red blood cells, and often after vascular ligature. It is a single center uncontrolled retrospective case series with no control group and limited generalizability. The authors used non-resorbable sutures, though removed early, which may still carry theoretical risks if removal is delayed. This is the first article using non-resorbable sutures [25].

This retrospective descriptive case series study took place in Thailand from January 2009 to December 2017 and included 57 women with uterine atony during cesarean section who did not respond to medical treatment. This new technique is a uterine flexion suture derived from the classic B Lynch uterine compression suture. The suture material isn’t specifically described; the only detail provided is that a standard absorbable suture was used to hold the uterus in an anteflexed position by combining vertical and horizontal components, without deeply penetrating the uterine cavity or requiring reopening of the hysterotomy in most steps. After exteriorization of the uterus, the suture starts on the right side of the cesarean incision, 1–2 cm below the lower edge and 3–4 cm from the lateral border, and exits at the upper edge of the incision on the left side, 1–2 cm above the incision and 3–4 cm from the left lateral border. The suture is passed without piercing the posterior wall, brought anteriorly and vertically over the fundus, then passed to the posterior wall at the same level. It crosses the midline horizontally on the posterior surface at a depth of 1–2 cm, then is brought back over the fundus to the anterior surface on the left side, mirroring the right side, and passed through the cesarean incision. An assistant holds the uterus in anteflexion, and the knot is tied while maintaining moderate tension. Maternal age ranged from 15 to 44 years, with 59.6% of the pregnant women (34 cases) being primiparous. The main indication for cesarean section birth was cephalopelvic disproportion in 47.4% of cases, and 19.3% had a previous cesarean section performed. The total number of deliveries during the study period was 37,843, with 44.1% cesarean deliveries; 57 cases experienced uterine atony and were treated with uterine flexion suture. The method proved to be efficient in all cases, with no postoperative excessive bleeding or hysterectomy. Moreover, no cases of serious postoperative complications were described, with ultrasound follow-up on days 1, 7, and 30 post-interventions. The work includes a small number of cases during a long study period. It also lacks detailed reports on the suture material and long-term fertility outcomes and complications, standardized objective measurements of blood loss, and is marked by potential selection bias [26].

D. Newer types of non-removable UCS

An article by Li D et al. (2025) [27], published in Frontiers in Medicine, describes a newer type of UCS. It is a retrospective analysis of a case series from a single-center hospital in China, conducted from December 2014 to December 2023, and included 45 patients with refractory PPH who underwent uterine mesh compression suturing. The authors did not provide the total number of deliveries or cesarean sections during the 10-year period, making it impossible to estimate PPH incidence. Postoperative PPH was defined as bleeding exceeding 1500 mL within 24 h after the cesarean section, caused by uterine atony and failure of conservative measures. They excluded birth canal injuries, coagulation disorders as the primary cause of PPH, and placenta accreta spectrum. They present an innovative technique using uterine mesh compression suturing with 2–3 vertical mattress sutures that pass through the full thickness of both the anterior and posterior uterine walls, creating a three-dimensional mesh compression effect, but without explicitly mentioning the suture material. The first suture starts on the anterior lower uterine segment, 3 cm from the incision edge and 3 cm from the left side. It passes full thickness through the uterine cavity to the posterior wall and runs longitudinally upward toward the uterine fundus. It goes over the fundus and continues to the posterior wall, then turns horizontally to the right side. Afterwards, the right side is mirrored symmetrically. The second suture is placed 3 cm above and parallel to the first suture, following the same path. If needed, a third suture is added, particularly if the uterine body is over 9 cm wide. The sutures are tightened while the uterus is exteriorized, and the final knot of the first suture is tied at the lower uterine segment after closing the cesarean incision. The sutures form a crisscross, circumferential, mesh-like network that applies uniform, balanced compression over a large area of the uterus, following the natural direction of uterine involution and compressing the arcuate arteries of the myometrium. The declared success rate was 100%, with no cases of hysterectomy performed. The bleeding stopped within 10 min after completing the mesh suturing, and the cumulative vaginal bleeding volume within the first 2 h after the procedure was less than 20 mL. The authors didn’t register any postoperative infections or uterine necrosis. The follow-up was between 6 months and 5 years, with menstruation resumed in all included women, no intrauterine adhesions (21 cases had diagnostic hysteroscopy performed), 10 women had subsequent pregnancies, 4 of them delivered by repeated cesarean section without complications, and 6 had induced abortions. It is a single-center observational study, without any control group, with limitations acknowledged by the authors (retrospective design, small sample size, short follow-up in some cases). The highlighted advantages of this technique compared to traditional UCS are uniform three-dimensional compression, better preservation of uterine cavity drainage, a lower risk of infection and necrosis, and rapid hemostasis [27].

E. UCS complication articles

Also, the literature includes several case reports of uncommon UCS complications, which we included in Table 3 [28,29,30,31,32,33,34,35,36,37,38].

F. Future fertility outcomes

Although focused primarily on reproductive outcomes after uterine-sparing procedures for PPH, three observational studies provide complementary long-term data that help complete the literature on PPH [39,40,41]. Rasheed et al. [39] conducted a multicenter cross-sectional study across five Egyptian university hospitals from 2008 to 2012, with results published in 2014. They ultimately analyzed 166 cases among 293 initially identified women with PPH managed with conservative surgical measures. They were divided into an IIAL group comprising 59 cases, a stepwise uterine devascularization group with 65 cases, and a B Lynch UCS group with 42 cases. The authors report novel associations, including a higher endometriosis rate after B Lynch sutures and reduced ovarian reserve after stepwise devascularization [39]. The 2014 Fuglsang [40] work reviewed a Danish registry of 43 classical B Lynch procedures performed in 42 women between 2002 and 2012, with a mean follow-up of 45 months. They reported two women with early postoperative complications, including menorrhagia, fever, and retained products of conception (residual placental tissue); one woman with Asherman’s syndrome; and one peripartum hysterectomy [40]. The third study is a larger single-center retrospective cohort study from Chicago, authored by Cowan et al. [41], with a 2000–2010-time frame. The authors identified 220 B Lynch procedures among 4800 cases of PPH and compared 63 pregnancies with a control group of 189 women who had experienced PPH without B Lynch sutures. After multivariable adjustment, the study found no significant association between prior B Lynch UCS placement and adverse outcomes in the next pregnancy, but the results remain limited [41].

## 4. Discussion

Because comparative studies were scarce, observational studies, case series, and case reports were included, and thus becoming methodologically inappropriate for a meta-analysis. Owing to substantial clinical and methodological heterogeneity, the findings were synthesized and stratified accordingly.

A. UC

Complications

Some studies address both short- and long-term complications, with particular attention to fertility preservation and menstrual function [8,12,13]. Others focus primarily on the efficacy of conservative surgical techniques for controlling PPH [11,14,15,17], although Kim et al. and Liu et al. briefly note four subsequent pregnancies among the nine women who desired further children [13,16].

In their 2023 study, Kwong et al. compared UCS alone with UCS plus additional hemostatic procedures: UAL or UE. The combined procedure group likely represented more severe PPH. Immediate complications were similar across groups and included puerperal pyrexia, wound infection, venous thromboembolism, secondary PPH, endometritis, and retained products of conception. Three cases of hematometra and one case of suture-strap erosion occurred, but without any reported uterine necrosis cases [11].

Kwong et al. (2023) [8] reported on a prospective cohort with short-term complications, including four cases of hematometra requiring drainage, four cases of endometritis treated with antibiotics, and one retained product of conception requiring surgical evacuation. Long-term menstrual and fertility outcomes included hypomenorrhea in eight women and Asherman’s syndrome in two. No infertility was reported among the 23 subsequent pregnancies. Compared with a matched control group of women managed solely with uterotonics for atonic PPH, the UCS group had higher rates of omental or bowel adhesions, recurrent atonic PPH, and the need for repeated compression sutures. The authors noted that Hayman and Cho sutures may carry a greater risk of endometrial scarring than B Lynch sutures because they compress the anterior and posterior walls more directly. They stressed the importance of appropriate suture tension and the use of firm, monofilament, rapidly absorbable material to minimize ischemic injury and scarring [8].

In contrast, Suzuki et al. described no severe complications after UCS placement but found that UCS performed during emergency cesarean section was associated with a higher incidence of postoperative endometritis [12]. Liu et al. described 12 uncomplicated UCS cases and reported an overall complication rate of 25% (one pyometra, one case of persistent vaginal discharge, one retained product of conception, and one case of endometritis) [13]. Nauman et al. reported minimal postoperative morbidity, including one wound infection, one episode of fever, two cases of mild abdominal pain, and only one peripartum hysterectomy for uncontrolled bleeding [15]. Kim et al., the only study restricted to twin cesarean deliveries, after excluding vaginal births and monochorionic monoamniotic twins, observed no UCS-related complications. The incidence of uterine atony was higher than in other series, likely reflecting the exclusive twin-pregnancy cohort [16].

Strengths

Compared with the broader UCS literature, including the same group’s 2020 work, Kwong et al. (2023) [8] present institutional PPH management guidelines and compare UCS outcomes with those of uterotonic-only management [8,10]. It reports estimated blood loss, parity, UCS type, suture indications, relevant maternal and fetal comorbidities such as hypertension and diabetes, and the underlying cause of PPH. It also provides long-term follow-up on fertility, menstrual recovery, and psychological impact, along with short-term complication data [8].

Limitations of the included studies

Like Suzuki et al., Liu et al., Charoendumrongsup et al., Nauman et al., Kim et al., and Latif et al., included only cesarean section deliveries in which UCS was performed intraoperatively, 2023 Kwong et al. included mostly cesarean section births with only one vaginal delivery [8,12,14,15,16,17]. Kim et al. further restricted the cohort to twin pregnancies [16]. 2020, Kwong et al. [11] included predominantly cesarean section births, with only one vaginal delivery, whereas Latif et al. [17] reported inclusion of only emergency cesarean sections [11,17]. Liu et al. assessed UCS efficacy by retaining the three cases that ultimately required hysterectomy [13].

The concurrent use of UCS and UAL for placenta previa cases in 2023, as reported by Kwong et al., introduces heterogeneity relative to pure atony cohorts [8]. PPH incidence may have been overestimated in the 2023 Kwong study [8], which defined PPH as blood loss greater than 1000 mL after cesarean delivery, whereas Suzuki et al. used a threshold of greater than 1500 mL [8,12]. Liu et al. [13] provided no definition for PPH, whereas Nauman et al. [15], Latif et al. [17], and Kim et al. [16] adhered to conventional definitions, with blood loss greater than 1000 mL in cesarean sections. Charoendumrongsup et al. [14] clearly overestimated PPH, defining it as blood loss above 300 mL [11,13,14,15,16,17].

In the 2023 article by Kwong et al. [8], the control group of women successfully treated with uterotonics alone almost certainly experienced milder hemorrhage. None of the included studies employed a control group. Observed differences in adhesions, recurrent atonic PPH, and the need for repeat sutures may therefore reflect greater initial severity rather than a direct effect of the sutures. Hysteroscopy for suspected intrauterine synechiae was performed only in symptomatic women with hypomenorrhea, likely underestimating mild, asymptomatic cases. Menstrual and psychological outcomes were not compared with the control group, further limiting interpretation [8].

In most studies, the population consisted largely of young, primiparous women undergoing cesarean delivery for singleton pregnancies. Results may therefore not be generalizable to multiparous women or to vaginal deliveries [11,16]. By contrast, Liu et al. enrolled roughly equal proportions of primiparous and multiparous women but did not report the overall incidence of PPH or its definition [13]. In both Nauman et al. [15] and Latif et al. [17], the high percentages of multiparous cases enrolled, 82.5% and 76.8%, respectively, underscore multiparity as a well-known PPH risk factor [15,17]. All authors reported the type of suture material, though there were variations depending on the UCS type. Kwong et al. used No. 1 Monocryl for B Lynch and Hayman sutures and No. 1 Vicryl for both Cho suture and UAL [8,11]. No. 1 Vicryl was used by Liu et al. and Kim et al. [13,16], whereas Nauman et al., Latif et al., and Charoendumrongsup et al. used No. 1 or No. 2 chromic catgut when performing UCS [14,15,17].

Compared with the 2023 Kwong et al. research and the work of Suzuki et al., the remaining seven studies by Liu et al., Charoendumrongsup et al., 2020 Kwong et al., Kim et al., Nauman et al., and Latif et al. constitute level IV evidence [8,11,12,13,14,15,16]. Their retrospective designs predispose them to selection bias, and single-center recruitment, with predominantly cesarean deliveries, limits generalizability. Several authors omitted the total number of births in the study period, the number of pharmacologically managed PPH cases, and clear, homogeneous definitions and the incidence of PPH. Most studies lack data on estimated blood loss, hemoglobin drop, transfusion requirements, or coagulation parameters at the time of surgery, hindering adjustment for baseline severity [13,14,17]. Excluding hysterectomy cases from the analysis may overestimate UCS efficacy and underestimate severe complications [8,12]. No adjustment for important confounders was performed in most cases [13,14,15,16,17].

B. Uterine Compression Sutures vs. Bakri balloon tamponade, UAE, or other conservative surgical methods

All four studies included in this section, which compared nonpharmacological surgical techniques for PPH management, are limited by small sample sizes, single-center experience, incomplete adjustment for severity, and scarce long-term fertility or menstrual data [18,19,20,21].

The four included studies evaluate various uterus-sparing approaches for PPH. Although all studies aimed to control hemorrhage while avoiding hysterectomy and preserving reproductive potential, direct comparison of their results is limited by significant differences in study design, PPH definition, patient selection, hemorrhage severity, causes of PPH, timing of the intervention, and availability of additional procedures. The efficacy of the method appears to depend strongly on the severity and causes of bleeding, particularly on the promptness of the intervention.

PPH incidence was reported in only two of the four studies. Puangsricharoen et al. reviewed 30,271 deliveries and identified 669 cases of PPH, corresponding to a 2.2% PPH incidence [20]. The definition of PPH followed guidelines, with more than 500 mL after vaginal delivery or greater than 1000 mL after cesarean delivery. Of the 669 included women, only 61 (9.1%) failed medical treatment and subsequently required conservative interventions or hysterectomy [20]. Similarly, Klangsin et al. reported 1461 cases of PPH among 39,327 deliveries, corresponding to an incidence of 3.7%. In this study, early PPH was defined as bleeding occurring within 24 h after delivery. However, PPH was identified clinically when medical treatment failed, rather than through a standardized quantitative blood loss measurement. These differences in reported incidence rates should therefore be interpreted cautiously. This methodological heterogeneity limits comparison with the Cairo and Turkish studies, which also focused on selected women with severe or medically refractory PPH rather than reporting the incidence of PPH among all deliveries [18,19].

The Cairo randomized clinical trial provides the strongest comparative evidence among the four studies because it prospectively randomized women with uncontrolled primary PPH to UAE or surgical management. 23 women were ultimately analyzed, with 11 undergoing UAE and 12 undergoing stepwise devascularization with compression sutures. The UAE group achieved technical success in 9 of 11 cases (81.8%), while 2 developed DIC and required hysterectomy. In the surgical group, 3 of 12 women required hysterectomy. Thus, the hysterectomy rates were 18.2% after UAE and 25% after surgical devascularization plus B Lynch. Although the authors described a fivefold reduction in hysterectomy with UAE, the small sample size results in very low statistical certainty. Essential radiologic parameters such as embolization agents, vascular recanalization rates, post-embolization syndrome, and imagistic surveillance of post-suture uterine ischemia are omitted in Farouk et al. research [18]. Kaya et al. offer a direct retrospective comparison between B Lynch UCS and Bakri balloon tamponade in 45 women with uterine atony during cesarean section. The success rates were similar for both methods, 79.1% and 80%, with the additional use of IIAL increasing the rates to 91.6% for B Lynch and 95% for Bakri balloon tamponade. No differences in estimated blood loss, transfusion requirements, time to control bleeding, or hospital stay were observed, supporting the concept that the initial method choice might be less important than timely escalation when the first conservative method fails. The Turkish authors emphasize the practical advantages of UCS in low-resource settings because of its rapid application, low cost, and lack of requirement for additional equipment, whereas the Bakri balloon is less invasive and easier to learn but requires additional equipment and may be more expensive [19]. The Turkish authors’ conclusion should not be interpreted as evidence that IIAL is universally preferable, because it is technically more demanding and may carry vascular and ureteric complications despite the observed increased success rate.

The Klangsin et al. [21] study represents a substantially larger experience. Among 39,327 deliveries, 92 women required conservative surgical management after medical therapy failure, including B Lynch UCS, Bakri balloon tamponade, uterine/ovarian/internal iliac artery ligation, combined vessel ligation, and other combinations. The overall initial success rate was 73.9%, increasing to 80.4% after additional procedures, with 19.6% of women ultimately requiring hysterectomy. B Lynch UCS was the most frequently used technique and had a success rate of approximately 71% when used alone [21]. The consistency between the Turkish and Thai studies is notable, with B Lynch alone having a success rate of around 70–80%, whereas combining UCS or balloon tamponade with vascular ligation increased the likelihood of successful uterine preservation. Nevertheless, the heterogeneous use of combined procedures makes it difficult to attribute the final outcome to a single technique [19,20].

The amount of blood loss varied considerably across studies and was an important determinant of outcome. Puangsricharoen et al. reported mean estimated blood loss of 3346 ± 2647 mL in the Bakri group, 2984 ± 2540 mL in the B Lynch group, and 2133 ± 1001 mL in the UAL group, indicating severe hemorrhage [20]. Klangsin et al. reported a median blood loss of 1800 mL before conservative surgery [21]. In this study, a shorter interval between diagnosis and initiation of conservative treatment, younger maternal age (<30 years), and blood loss < 1800 mL was independently associated with higher success rates [20]. In contrast, the Turkish study found no significant differences in estimated blood loss between the B Lynch and Bakri groups, suggesting that neither technique was clearly superior as the initial conservative procedure. However, the study did not provide a standardized blood loss protocol, limiting the precision of this comparison [19].

These findings provide an important explanation for differences in success rates across studies. Conservative techniques are more likely to succeed before profound blood loss, coagulopathy, and DIC develop. Once massive hemorrhage and coagulopathy are established, mechanical compression or vascular occlusion may be less effective, and hysterectomy may become necessary.

Postoperative morbidity was particularly notable in the Thai studies. Puangsricharoen et al. reported DIC in 25.8% of 31 women undergoing conservative treatment. DIC occurred in five women in the Bakri group and three in the B Lynch group. Bladder and bowel injuries were also reported, but these occurred during cesarean delivery and cannot necessarily be attributed directly to the conservative PPH techniques. One patient required massive transfusion and developed pulmonary edema. No maternal deaths occurred [20]. Klangsin et al. reported an early postoperative complication rate of 27.2%. Complications were predominantly associated with hemorrhage severity and included massive hemorrhage, DIC, pulmonary edema, and acute renal failure, with no reported maternal deaths [21]. The Cairo randomized trial reported DIC in both treatment groups, with 2 of 11 women in the UAE group and 3 of 12 in the surgical group. However, the study was too small to provide reliable estimates of comparative complication rates [18]. Overall, reported complications appear to be influenced more by the severity and duration of hemorrhage than by the choice of conservative technique alone. This is supported by the Klangsin et al. study, in which earlier interventions and lower blood loss were associated with successful treatment [21].

Preservation of fertility is a principal advantage of conservative management over hysterectomy. The most informative data in this regard come from Puangsricharoen et al. [20]. All 23 women who had successful conservative treatment resumed normal menstruation. Of the 11 women who desired a subsequent pregnancy, three conceived viable pregnancies within one year: one of four women treated with Bakri balloon tamponade, one of 5 B Lynch UCS, and one of two UAL cases [20]. These results are encouraging but should not be interpreted as evidence of reduced fertility caused by the procedures, given the small sample size and the lack of desire for a subsequent pregnancy in most women. Furthermore, the study relied on telephone follow-up to assess subsequent pregnancy outcomes, without a comprehensive assessment of reproductive status [20]. The Cairo study did not provide sufficient long-term fertility follow-up to permit meaningful comparison of reproductive outcomes between UAE and conservative surgical interventions [20]. Similarly, the Turkish study did not provide detailed long-term fertility or menstrual follow-up [19]. These limitations are important because uterine preservation does not necessarily result in preserved reproductive function. B Lynch sutures may also have specific long-term concerns, including intrauterine or abdominal adhesions, although the clinical impact of these findings remains uncertain [18,19,20,21]. Therefore, long-term menstrual and reproductive follow-up should be considered particularly important when fertility preservation is a primary objective.

The duration and quality of post-intervention follow-up varied substantially across the studies. Puangsricharoen et al. specifically assessed postoperative complications at six weeks and subsequently contacted patients regarding pregnancy outcomes, providing the most informative follow-up among the four studies. Nevertheless, the number of women attempting a subsequent pregnancy was very limited [20]. Farouk et al.’s randomized trial had the major methodological advantage of randomization, reducing selection bias, but its sample size was extremely small. In addition, it included only women with atonic PPH, limiting applicability to other etiologies such as placenta accreta. The online study also did not provide sufficient long-term fertility outcomes [18]. Kaya et al. provided a useful direct comparison between two commonly used techniques but was retrospective, non-randomized, and single-center. Treatment selection was influenced by clinical judgment, and the small sample size limited its statistical power. It also focused on uterine atony PPH during cesarean birth, limiting its generalizability [19]. The Puangsricharoen et al. study similarly had a retrospective design and a very small sample size, particularly in the UAL group. Treatment selection depended on patient preference and surgeon judgment, creating a substantial risk of selection bias. Moreover, the mean blood loss was high and heterogeneous, and no randomized comparison was possible [20]. Klangsin et al. provided the largest cohort and the most useful information regarding predictors of success, including maternal age, blood loss, and time to intervention. However, the study was retrospective and single-center, used clinically estimated rather than quantitatively measured blood loss, lacked a standardized treatment protocol, and included different combinations of uterine conservative procedures. Consequently, the reported 80.4% success rate should not be interpreted as the efficacy of one specific technique [21].

Taken together, these studies support uterine-sparing treatment as an effective strategy for failed medical management of PPH. B Lynch, UCS, and Bakri balloon tamponade demonstrated broadly comparable success rates in Kaya et al.’s research [19], while the Thai study underscores that success rates can increase when additional vascular ligation or other conservative procedures are added [20]. The most consistent finding across these studies is that timing and the severity of hemorrhage may be more important determinants of successful uterine preservation than the choice among different conservative techniques. B Lynch has many advantages, including speed, simplicity, low cost, and applicability in resource-limited settings, whereas Bakri balloon tamponade offers a less invasive approach and is also relatively easy to learn. UAE provides a minimally invasive alternative when an interventional radiology service is immediately available and the patient is sufficiently stable for transfer and embolization. Stepwise devascularization and IIAL remain valuable surgical options, particularly where UAE is unavailable, although IIAL requires greater surgical expertise [18,19,20,21].

C. Newer types of removable and non-removable UCS

Five studies described innovative removable UCS, and one presented an innovative non-removable UCS [22,23,24,25,26]. In 2018, Li et al. [22] reported a retrospective series of 12 women with refractory PPH during cesarean section who were managed with a novel removable utero-pubic ring compression suture. The three failure cases were managed by UAL, with no hysterectomy performed [23]. In 2015, Zhang et al. [24] described removable modified B Lynch and Hayman sutures, performed with No. 2 Polyglactin and extracted vaginally after a mean of 21.6 h, in a very small series of five cases, with a 100% success rate and no immediate complications [24]. The first study to present a newer type of removable UCS was the 2014 Aboulfalah et al. study, which introduced a non-absorbable brace suture into clinical practice for 15 Moroccan women with severe blood loss. The suture was removed percutaneously within 24–48 h, achieving 100% initial efficacy, although one secondary hysterectomy and one unrelated maternal death occurred [25]. The 2018 Songthamwat et al. article evaluated a uterine flexion suture, a modified B Lynch suture performed with absorbable material, that maintained the uterus in anteflexion without deep cavity penetration in 57 Thai women with cesarean atony, reporting a 100% success rate and no hysterectomies or serious complications [26]. The only newly described non-removable UCS was reported by Li et al. in 2025 [27]. They reported a series of 45 Chinese women with post-cesarean refractory PPH who were treated with two or three full-thickness vertical mattress sutures to create a uterine mesh over the uterine body, achieving a 100% success rate with no complications [27].

D. Future fertility outcome

Although focused on reproductive outcomes after UCS, the cross-sectional multicentric observational study conducted by Rasheed et al. in 2014 [39] displays information of great importance. They followed the evolution of women who underwent uterine-sparing procedures for PPH from five university hospitals in Egypt over a four-year period from September 2008 to September 2012. They initially included 293 cases, and the final analysis comprised 166 cases after exclusions and losses to follow-up. They divided the cases into four groups: the first included 59 women with bilateral iliac artery ligation; the second included 65 women with stepwise devascularization (SWUD); the third, which included only two women with bilateral uterine artery ligation, was further excluded from the analysis due to the small number of cases; and the last comprised 42 women with B Lynch compression sutures. The authors do not specify the type of Vicryl used for the sutures performed. The incidence of PPH, the rate of conservative surgical interventions, and the definition of PPH were also not mentioned in the article. The study did not directly evaluate the acute efficacy of hemostatic sutures (their success rate, hysterectomy avoidance, blood loss), focusing only on long-term outcomes. It is a level III evidence cross-sectional observational study with selection bias, as women were recruited from outpatient infertility and antenatal clinics and had fertility problems, with a high dropout rate. The study had no control group, with comparison made only between the three mentioned groups. It is very hard to establish a clear temporal/causal relationship between the procedure itself and the women’s fertility outcome. The mode of birth was also not specified, nor were the causes of PPH or parity. The novel findings reported, a high endometriosis rate after B Lynch sutures and reduced ovarian reserve after SWUD, require confirmation from better-designed studies [39]. Despite being one of the largest long-term follow-up studies of reproductive outcomes after uterine-sparing procedures, it has several methodologic limitations, including selection bias, lack of controls, and missing key data.

A retrospective registry-based study with medical record review and follow-up from Denmark, conducted from January 2002 to March 2012, with follow-up data collected until July 2013 describes a total of 43 classical B Lynch procedures performed in 42 women (one woman had B Lynch sutures in two subsequent pregnancies), with no specification of the suture material. The health facility registered approximately 4500–5000 deliveries per year. The mean fertility follow-up was 45 months, ranging from 17–126 months. Two women reported postoperative complications, including menorrhagia, fever, and residual placental tissue; one case of Asherman’s syndrome; and one peripartum hysterectomy. It is a level 4 small retrospective study with one case lost to follow-up, no control group, potential underreporting of complications, and limited detailed obstetric outcome data in subsequent pregnancies. No specific incidence of PPH was reported, consistent with its low evidence level IV status as a small retrospective follow-up study with registry data. Of the 42 included women, 16 (38%) became pregnant at least once after the B Lynch procedure. Of these, two had very early induced abortions in the first trimester, and one had a termination at 7 weeks due to a scar pregnancy, leaving 14 that progressed beyond the first trimester. No information was provided on the exact final number of live births from those 14 pregnancies. The design is retrospective, with potential selection and information bias, and no control group. Of the 26 primiparous cases included in the study who needed B Lynch sutures, 44% had a subsequent pregnancy or gave birth to their second child by the end of the follow-up period [40].

A retrospective cohort study conducted at Northwestern Memorial Hospital, a large tertiary care center in Chicago, Illinois, USA, from January 2000 to June 2010 included 220 women who underwent B Lynch suture placement for PPH. Of these, 63 had subsequent pregnancies at the same institution and were included in the analysis. A control group of 189 women with PPH in the index pregnancy but without B Lynch sutures was selected for comparison. The suture material used for the B Lynch UCS was not specified in the article. The total number of PPH cases during this period was 4800, and 4.6% (220 cases) were treated with B Lynch sutures. The article does not provide information about the number of deliveries from this institution. PPH was defined as occurring in pregnancies reaching 24 weeks of gestation, without any specific blood loss volume or clinical criteria. The focus was on subsequent pregnancies rather than the hemostatic success of this type of UCS. After multivariable adjustment for potential confounders, there was no significant association between prior B Lynch suture placement and an increased risk of adverse outcomes in the next pregnancy. The study has a moderate level III of evidence, being a retrospective cohort study with a control group and multivariable adjustment. The retrospective design has an inherent risk of selection bias, and the single-center experience limits generalizability. No data on B Lynch procedure complications or its success rate were reported. There is also no information regarding the suture material or surgical technique variations. The control group included women with PPH without B Lynch compression sutures, but the groups might differ in bleeding severity, with no details about how bleeding was managed in those cases. The authors report a mean estimated blood loss in the control group ranging from 1200 to 1800 mL [41].

The predominance of retrospective, uncontrolled studies may overestimate treatment success through selection bias, incomplete ascertainment of complications. Confounding by indication is also likely because patients with more severe hemorrhage were more likely to receive combined or rescue procedures. Consequently, lower success proportions cannot necessarily be attributed to an inferior technique, while outcomes following combined interventions cannot be attributed to UCS alone. Conventional B Lynch sutures have the largest body if clinical experience, but the evidence does not establish their superiority over other techniques. Removable sutures are designed to avoid permanent cavity compression, and may theoretically offer the advantage of reduced adhesions and more favorable fertility outcomes. But the techniques are evaluated only in small, uncontrolled, single-center series. They can be described as promising, but without any proof of their superiority compared to traditional UCS techniques.

Our review has significant limitations. The most important ones reside in the included scientific literature, with mostly retrospective, small case series, single centered, with no randomization, control group, or adjustment for confounders. Our research was also restricted to only three electronic database and did not include grey literature database, which may have introduced publications and retrieval bias. Our broad inclusion criteria also increased the methodological heterogeneity, making it difficult to synthesize our conclusions based on the results of the included articles. Our conclusions remain highly dependent on the quality and completeness of the underlying evidence.

## 5. Conclusions

USC are fertility-preserving surgical options for refractory PPH. Most research originates from Asia (Japan, Hong Kong, Singapore, Korea, Thailand, Pakistan), reflecting experience at tertiary referral centers that typically manage more severe cases of PPH. Asian tertiary centers dominate the literature and report good uterine preservation rates, but retrospective design, small samples, lack of controls, inadequate adjustment for confounders, and heterogeneous techniques limit strong conclusions and generalizability. The predominance of cesarean section cohorts reduces generalizability to vaginal births. Long-term risks such as adhesions, synechia, and hypomenorrhea appear more frequent with certain suture types, such as Hayman and Cho’s, and in severe cases.

Reported uterine preservation, as equivalent to the techniques success rate, ranges in the selected articles from 73.9% to 100% in single-technique series, but appears strongly influenced by bleeding severity, and case complexity: success rates were consistently higher for isolated atonic PPH managed with UCS alone and lower when additional hemostatic procedures were required, reflecting the more severe cases typical of large referral hospitals. The certainty of the available evidence remains low, with the estimates being unavailable for a direct comparison due to heterogeneity in PPH definition and severity, patient selection, outcome definitions, concomitant interventions, and follow-up. The B Lynch suture has the broadest clinical experience and may be particularly applicable where rapid surgical control is required and interventional radiology is unavailable. Removable and modified UCS techniques may offer theoretical safety advantages, but current evidence is limited to small, uncontrolled series.

This body of evidence includes real-world experience predominantly from high-volume Asian centers, relatively large cumulative case numbers for a rare intervention, and some focus on long-term menstrual and fertility outcomes, with Kwong et al.’s 2023 [8] work being one of the stronger prospective contributions.

Larger prospective multicenter studies with standardized protocols are needed to better define optimal suture selection based on bleeding severity and hospital setting. Longer-term prospective follow-up of menstruation, uterine anatomy, intrauterine adhesions, ovarian function, conception, pregnancy complications, and live-birth outcomes is needed. Future studies need to adopt a standardized PPH definition, use quantitative blood-loss measurement, define treatment algorithms clearly, and include larger comparative cohorts to determine whether one uterine-sparing technique provides a clinically meaningful advantage over another. Studies should compare predefined UCS techniques using standardized primary endpoints, including primary hemostatic control without additional procedures and hysterectomy-free survival. Secondary endpoints should include transfusion requirements, reoperation, infection, uterine ischemia or necrosis, intrauterine synechiae, menstrual function, conception, pregnancy complications, and live-birth outcomes. Follow-up intervals and imaging protocols should also be standardized.

## Figures and Tables

**Table 1:** Search queries.

Database	Search Terms	Filters Applied	Date Searched	Hits
PubMed	((postpartum) AND (hemorrhage)) AND ((hysterectomy) OR (B-Lynch suture) OR (Cho suture) OR (Hayman suture) OR (artery ligation) OR (compression suture))	2014–2025	28 April 2026	1531
Web of Science	((postpartum) AND (hemorrhage)) AND ((hysterectomy) OR (B-Lynch suture) OR (Cho suture) OR (Hayman suture) OR (artery ligation) OR (compression suture))	2014–2025	28 April 2026	2204
Cochrane Library	((postpartum) AND (hemorrhage)) AND ((hysterectomy) OR (B-Lynch suture) OR (Cho suture) OR (Hayman suture) OR (artery ligation) OR (compression suture))	2014–2025	28 April 2026	58

**Table 2:** Overview of level IV retrospective case series included in the review.

No	Article	Year of Study	No of Births	No of Included Cases	Type of Birth	Definition of PPH (Yes/No)	Mean/Median Maternal Age	Multiparity	Surgical Material	Type of Suture	Succes Rate UCS	Singleton Pregnancies	Bleeding Cause	Cofounder Variables	Follow-Up	No of Hysterectomies	Complications	Blood Loss Quantity
1	Liu et al., 2014 [13]	2008–2012	59,665	23	Caesarean section	No	31.5 (mean)	47.8%	No. 1 Vicryl	Mostly B Lynch	83%	Yes	21 cases uterine atony	Results not adjusted to cofounders	Yes	4 cases—3 due to persistent bleeding, and one due to pyometra	4 cases with persistent vaginal discharge, pyometra, retained product of conception and endometritis	NM
2	Kim et al., 2020 [16]	2013–2018	953	147 UCS/806 without UCS	Caesarean section	Yes (>1000 mL with signs of hypovolemia)	34.0 ± 3.1	8.8%	No. 1 Vicryl	Modified B Lynch or Cho’s suture	98.60%	Only twin pregnancies delivered by cesarean section after 24 weeks of gestation; Monochorionic, monoamniotic twin pregnancies and vaginal deliveries were excluded	Uterine atony with or without placenta previa or accreta	Results not adjusted to cofounders	Yes—2 months	0 (2 cases required additional UAL)	0	725.2 ± 256.1 mL
3	Kwong et al., 2020 [11]	2009–2019	60,714	79	78 caesarean sections, 1 vaginal birth	Yes (1000 mL or more, or bleeding leading to symptoms or signs of hypovolemia within 24 h of birth regardless of delivery route)	32 (median)	16.5%	No. 1 Monocryl for B Lynch and Hayman’s sutures; No. 1 Vicryl for Cho’s suture; No. 1 Vicryl for UAL	B Lynch, Hayman’s and Cho’s sutures	97.70%	16.5% had multiple pregnancies	Uterine atony Placenta previa and/or accreta (7 cases) Amniotic fluid embolism (1 case)	Results not adjusted to cofounders	Yes—6 weeks for complications	10 (43 UCS cases with only one case needing hysterectomy)	puerperal pyrexia (3), venous thromboembolism (1), secondary PPH (5), readmission after initial discharge (9), endometritis (6), hematometra (3) or retained products of conception (4), with no case of uterine necrosis/erosion or pyometra reported in UCS	UCS only—2500 mL (1600–3200 mL); Uterine compression suture with additional hemostatic procedures—4100 mL (3075–5225 mL)
4	Nauman et al., 2020 [15]	2017–2018	932	932 caesarean sections	40 caesarean sections	Yes > 1000 mL	67.5% between 20–30 years 22.5% > 30 years old and 10% < 20 years old.	82.5%	No 1 Chromic catgut	Hayman suture	97.5%	Yes	Uterine atony	Results not adjusted to cofounders	NM	1	fever in 3 women, 2 mild abdominal pain, and 1 infected wound	22 cases between 1000–1300 mL; 11 cases between 1301–1600 mL and 7 cases between 1601 and 2000 mL
5	Latif at al., 2022 [17]	2020–2021	NM	314	Emergency caesarean section	Yes > 1000 mL	Mean 27.46 ± 5.61	76.8%	No. 2 chromic catgut	B Lynch	95.9%	Yes	NM	Age and parity stratification	NM	13	NM	NM
6	Charoendumrongsup et al., 2018 [14]	2013–2014	7500 deliveries with 3375 cesarean sections	50	Cesarean section	Yes > 300 mL	Mean 27.4 ± 6.3	Yes but percentage not mentioned	No. 1 chromic catgut	B Lynch	94%	7 twin pregnancies	Uterine atony	Results not adjusted to cofounders	Yes—6 months	0	3 cases had postoperative postpartum hemorrhage managed by using uterotonics	Between 300–1500 mL

**Table 3:** Case presentations of UCS complications.

No	Author/ Year	Suture Type	Complication	The Cause for PPH	Suture Material	Country	Case Particularity
1.	Takeda et al. 2019 [28]	VCS	Consecutive uterine ischemia with pelvic pain	A combination of placenta praevia and uterine atony	NM	Japan	First reported laparoscopic removal of vertical compression sutures
2.	Benkirane et al. (2017) [29]	B Lynch + VCS	Uterine necrosis	Uterine atony	absorbable suture	Morocco	Combination of compression suture + triple vascular ligation led to necrosis
3.	Sebri et al. (2023) [30]	UCS + Artery ligation	Uterine necrosis + Sepsis-associated encephalopathy (revealing HIV)	Uterine atony	NM	Tunisia	Rare case revealing undiagnosed HIV infection
4.	Vanwinkel et al. (2021) [31]	B Lynch + UAL	Placenta accreta & uterine inversion in subsequent pregnancy (suspected)	Uterine atony	NM	Belgium	Long-term obstetric outcome after B Lynch
5.	Begum et al. (2014) [32]	B Lynch	Severe pelvic adhesions + uterine deformation + Secondary infertility	Massive PPH (placenta previa)	NM	India	Severe fertility impact 4 years later
6.	Moussaoui et al. (2020) [33]	B Lynch + Tsirulnikov triple ligation	Uterine necrosis + Septic shock	Uterine atony	Vicryl 1	Morocco	Required total hysterectomy
7.	Shen et al. (2025) [34]	B Lynch	Uterine necrosis with spontaneous endometrial prolapse	Uterine atony	NM	China	Unusual prolapse of necrotic endometrium
8.	Yang et al. (2025) [35]	Cross-binding suture + Bilateral UAL	Uterine necrosis + Hysterectomy	Intractable atonic PPH	NM	China	Cross-binding (unconventional) technique
9.	Feng et al. (2025) [36]	Modified B Lynch + Circumferential suture	Severe intrauterine adhesions + Upper endometrial necrosis	Uterine atony	No. 1 absorbable	China	Significant impact on future fertility
10.	Ferreira Jorge et al. (2024) [37]	B Lynch	Uterine necrosis (day 8)	Uterine atony	NM	Portugal	Required total hysterectomy
11.	Thakur et al. (2024) [38]	Modified B Lynch	Uterine necrosis	Uterine atony	braided suture	India	MRI features described

NM—not mentioned.

## Data Availability

The original contributions presented in this study are included in the article. Further inquiries can be directed to the corresponding author.

## Abbreviations

The following abbreviations are used in this manuscript:

PPH	Postpartum hemorrhage
mL	milliliters
ACOG	American College of Obstetricians and Gynecologists
RCOG	Royal College of Obstetricians and Gynecologists
WHO	World Health Organization
ICU	intensive care unit
UCS	uterine compression sutures
UAE	uterine artery embolization
VCS	vertical compression sutures
UAL	uterine artery ligation
IIAL	internal iliac artery ligation
SWUD	stepwise devascularization

## References

[1] Likis F.E., Sathe N.A., Morgans A.K., Hartmann K.E., Young J.L., Carlson-Bremer D., Schorn M., Surawicz T., Andrews J. (2015). Management of Postpartum Hemorrhage.

[2] Escobar M.F., Nassar A.H., Theron G., Barnea E.R., Nicholson W., Ramasauskaite D., Lloyd I., Chandraharan E., Miller S., Burke T. (2022). FIGO recommendations on the management of postpartum hemorrhage 2022. Int. J. Gynaecol. Obstet..

[3] Günaydın B. (2022). Management of Postpartum Haemorrhage. Turk. J. Anaesthesiol. Reanim..

[4] Committee on Practice Bulletins-Obstetrics (2017). Practice Bulletin No. 183: Postpartum Hemorrhage. Obstet. Gynecol..

[5] Mavrides E., Allard S., Chandraharan E., Collins P., Green L., Hunt B.J., Riris S., Thomson A.J., on behalf of the Royal College of Obstetricians and Gynaecologists (2017). Prevention and Management of Postpartum Haemorrhage: Green-top Guideline No. 52. BJOG.

[6] World Health Organization (2012). WHO Recommendations for the Prevention and Treatment of Postpartum Haemorrhage.

[7] Lindquist J.D., Vogelzang R.L. (2018). Pelvic Artery Embolization for Treatment of Postpartum Hemorrhage. Semin. Interv. Radiol..

[8] Kwong L.T., Wong S.F., So P.L. (2023). Menstrual, fertility and psychological impacts after uterine compression sutures for postpartum hemorrhage: A prospective cohort study. BMC Pregnancy Childbirth.

[9] Mlodawska M., Zmelonek-Znamirowska A., Swiercz G., Mehta J., Mlodawski J. (2026). Neonatal Respiratory Outcomes in Placenta Previa with Histopathologically Confirmed Placenta Accreta Spectrum. J. Clin. Med..

[10] Mlodawski J., Mlodawska M., Przybysz N., Bielak A., Detka K., Pasiarski M., Rokita W. (2021). Collection of um-bilical cord blood and the risk of complications in postpartum women after natural labor in the context of the possibility of umbilical cord stem cell use in clinical practice. Ginekol. Pol..

[11] Kwong L.T., So P.L., Wong S.F. (2020). Uterine compression sutures with additional hemostatic procedures for the management of postpartum hemorrhage. J. Obstet. Gynaecol. Res..

[12] Suzuki Y., Matsuzaki S., Mimura K., Kumasawa K., Tomimatsu T., Endo M., Kimura T. (2017). Investigation of perioperative complications associated with use of uterine compression sutures. Int. J. Gynaecol. Obstet..

[13] Liu S., Mathur M., Tagore S. (2014). Complications and pregnancy outcome following uterine compression suture for postpartum haemorrhage: A single centre experience. J. Obstet. Gynaecol..

[14] Charoendumrongsup P. (2015). B lynch suturing in uterine atony during ceasarean section. Reg. 11 Med. J..

[15] Nauman D., Saif N., Siddique U., Naveed N., Siddique U. (2020). Assessment of efficacy of Hayman suture technique in management of intraoperative atonic postpartum hemorrhage. Pak. J. Neurol. Surg..

[16] Kim M.L., Hur Y.M., Ryu H., Lee M.J., Seong S.J., Shin J.S. (2020). Clinical outcomes of prophylactic compression sutures for treatment of uterine atony during the cesarean delivery of twins. BMC Pregnancy Childbirth.

[17] Latif F., Qadir S., Aslam S., Gondal M., Arif R.M.H. (2022). Success rate of B Lynch suture in the treatment of postpartum hemorrhage in women undergoing emergency caesarean section. JAMDC.

[18] Farouk O., Elbasuony W., Elbohouty A. (2016). Uterine artery embolization versus surgical management in primary atonic postpartum hemorrhage: A randomized clinical trial. Egypt. J. Radiol. Nucl. Med..

[19] Kaya B., Guralp O., Tuten A., Unal O., Celik M.O., Dogan A. (2016). Which uterine sparing technique should be used for uterine atony during cesarean section? The Bakri balloon or the B Lynch suture?. Arch. Gynecol. Obstet..

[20] Puangsricharoen P., Manchana T. (2019). Conservative surgical management for immediate postpartum hemorrhage. Asian Biomed. (Res. Rev. News).

[21] Klangsin S., Booncharoen P., Suwanrath C. (2022). Conservative Surgical Management of Early Postpartum Hemorrhage: A 12-Year Experience in a Tertiary Care Center in the South of Thailand. J. Obstet. Gynaecol. India.

[22] Li G.T., Li X.F., Zhang Y.H., Si Y., Li G.R., Xu H.M. (2018). Ring compression suture for controlling post-partum hemorrhage during cesarean section. J. Obstet. Gynaecol. Res..

[23] Li G.T., Li X.F., Li G.R., Wu B.P., Zhang X.L., Xu H.M. (2021). Removable retropubic uterine compression suture for controlling postpartum hemorrhage. J. Obstet. Gynaecol. Res..

[24] Zhang Z.W., Liu C.Y., Yu N., Guo W. (2015). Removable uterine compression sutures for postpartum haemorrhage. BJOG.

[25] Aboulfalah A., Fakhir B., Ait Ben Kaddour Y., Asmouki H., Soummani A. (2014). A new removable uterine compression by a brace suture in the management of severe postpartum hemorrhage. Front. Surg..

[26] Songthamwat S., Songthamwat M. (2018). Uterine flexion suture: Modified B Lynch uterine compression suture for the treatment of uterine atony during cesarean section. Int. J. Women’s Health.

[27] Li D., Wang C., Li L., Yang X. (2025). Uterine mesh compression suturing for refractory postpartum hemorrhage: A retrospective study of 45 cases. Front. Med..

[28] Takeda J., Takeda S. (2019). Management of disseminated intravascular coagulation associated with placental abruption and measures to improve outcomes. Obstet. Gynecol. Sci..

[29] Benkirane S., Saadi H., Serji B., Mimouni A. (2017). Uterine necrosis following a combination of uterine compression sutures and vascular ligation during a postpartum hemorrhage: A case report. Int. J. Surg. Case Rep..

[30] Sebri H., Halouani A., Masmoudi Y., Aloui H., Triki A., Amor A.B. (2023). Sepsis-associated encephalopathy and uterine necrosis revealing HIV infection after artery ligation and uterine compression sutures: Case report. Int. J. Surg. Case Rep..

[31] Vanwinkel S., Claes L., Van den Bosch T. (2021). Obstetrical outcome after B Lynch sutures and ligation of uterine arteries: A case report. Case Rep. Women’s Health.

[32] Begum J., Pallave P., Ghose S. (2014). B Lynch: A technique for uterine conservation or deformation? A case report with literature review. J. Clin. Diagn. Res..

[33] Moussaoui K.E., Zraidi N., Baidada A., Kharbach A. (2020). Uterine necrosis following uterine compressions in combination with vascular ligation during postpartum hemorrhage: A case report. Pan Afr. Med. J..

[34] Shen L., Cao Y., Gu N., Chen L., Meng Q., Dai Y. (2025). Postpartum uterine necrosis with spontaneous endometrial prolapse after B Lynch suture: A case report. Int. J. Gynaecol. Obstet..

[35] Yang S.Y., Liu Y., Zhou Z.J., Yan J.B., Li T., Cao B.Y., Li J.Q. (2025). A case report of uterine necrosis following cesarean section: Lessons learned from cross binding suture for refractory postpartum hemorrhage. Front. Med..

[36] Feng Y., Bi H. (2025). Severe Intrauterine Adhesions Following Modified B Lynch Procedure. Matern. Fetal Med..

[37] Ferreira Jorge I., Rodrigues S., Dias G., Aboim L., Dias E., Veríssimo C. (2024). Uterine Necrosis in the Context of B Lynch Compressive Sutures: Case Report. EC Gynaecol..

[38] Thakur S., Jhobta A., Surya M., Tomar A. (2024). MR imaging features of uterine necrosis following modified B Lynch sutures: A case report. Egypt. J. Radiol. Nucl. Med..

[39] Rasheed S.M., Amin M.M., Abd Ellah A.H., Abo Elhassan A.M., El Zahry M.A., Wahab H.A. (2014). Reproductive performance after conservative surgical treatment of postpartum hemorrhage. Int. J. Gynaecol. Obstet..

[40] Fuglsang J. (2014). Later reproductive health after B Lynch sutures: A follow-up study after 10 years’ clinical use of the B Lynch suture. Fertil. Steril..

[41] Cowan A.D., Miller E.S., Grobman W.A. (2014). Subsequent pregnancy outcome after B Lynch suture placement. Obstet. Gynecol..

